# Account of Two Instances of Uncommon Formation in the Viscera of the Human Body

**Published:** 1797

**Authors:** John Abernethy

**Affiliations:** Assistant Surgeon to St. Bartholomew's Hospital.


XI. *Account of two Instances of uncommon Formation in the Viscera of the Human Body. By Mr.* John Abernethy, *Assistant Surgeon to St. Bartholomew's Hospital.* Vide *Philosophical Transactions of the Royal Society of London, for the Year* 1793. Part I. 4to. London, 1793.

THE peculiarities of the first of the two cases described in this paper, consist in an uncommon transposition of the heart, and distribution of the blood vessels; together with a very remarkable, and, perhaps, singular formation of the liver. The body, which contained these deviations from the usual structure, was brought to Mr. Abernethy for dissection; with its history whilst alive he is, he observes, unacquainted. The subject, we are told, was a female infant, which measured two feet in length; the umbilicus was firmly cicatrized, and the umbilical vein closed: from these circumstances our author concludes that it was about ten months old. The muscles of the child, which were large and firm, and covered vered by a confiderable quantity of healthy fat, together with the general appearance of the body, ftrongly implied, it feems, that the child had, when living, poffeffed much vigour of conftitution.

Mr. Abernethy firft relates thofe varieties of the fanguiferous fyftem which were found on the thoracic fide of the diaphragm; and afterwards defcribes thofe which were difcovered in the abdomen: this naturally leads him to the account of the uncommon ftate of the liver.

The fituation of the heart, he obferves, was reverfed; the bafis of that organ being placed a little to the left of the fternum, whilft its apex extended confiderably to the right, and pointed againft the fpace between the fixth and feventh ribs. The cavities ufually called the right auricle and ventricle were confequently inclined to the left fide of the body; therefore, to avoid confufion in the defcription, our author follows Winflow, in terming them anterior; whilft thofe cavities ufually called left, he terms pofterior. The inferior vena cava, we are told, paft, as ufual, through a tendinous ring in the right fide of the centre of the diaphragm; it afterwards purfued the courfe of the vena azygos, the place of which it fupplied: after having united with the ſuperior cava, the conjoined veins paſſed beneath the baſis of the heart, to expand into the anterior auricle. The veins returning the blood from the liver united into one trunk, which paſſed through a tendinous aperture in the left of the centre of the diaphragm, and terminated immediately alſo in the anterior auricle.

The diſtribution of blood to the lungs, and the return of it from thoſe bodies, were accompliſhed after the uſual manner.

The aorta, after it had emerged from the poſterior ventricle of the heart, extended its arch from the left to the right ſide, but afterwards purſued its ordinary courſe along the bodies of the dorſal vertebræ.

From the curvature of the aorta there firſt aroſe the common arterial trunk, which, in this ſubject, divided into the left carotid and ſubclavian arteries; whilſt the right carotid, and ſubclavian, proceeded from the aorta by diſtinct trunks.

The inferior aorta gave off the cæliac, which, as uſual, divided into three branches; but that artery which was diſtributed to the liver appeared, it ſeems, larger than common; and exceeded, by more than one-third, the ſize of the ſplenic

ſplenic artery of this ſubject. This was the only veſſel, our author obſerves, which ſupplied the liver with blood, for the purpoſe either of nutrition or ſecretion.

The vena portarum was formed in the uſual manner, but terminated in the inferior cava, nearly on a line with the renal veins. The umbilical vein ended in the hepatic vein.

The liver was of the ordinary ſize, but had not the uſual inclination to the right ſide of the body; it was ſituated in the middle of the upper part of the abdomen, and nearly an equal portion of the gland extended into either hypochondrium.

The gall bladder lay collapſed in its uſual ſituation; it was of a natural ſtructure, but rather ſmaller than common; and was found to contain a tea ſpoonful of a fluid which, in its colour and other properties, reſembled the bile of children.

The inteſtines did not contain much alimentary or fœcal matter; this was, however, we are told, as uſual, deeply tinged with bile.

The ſpleen conſiſted of ſeven ſeparate portions, to each of which a branch of the ſplenic artery was diſtributed. The other viſcera were found, and of their ufual ftructure and appearance.

Mr. Abernethy could difcover no caufe to which the child's death could be affigned. He obferved that the tongue was incrufted with a dark coloured mucus, which indicated the exiftence of fever previoufly to the infant's death.

When an anatomift, he obferves, contemplates the performance of biliary fecretion by a vein, a circumftance fo contrary to the general economy of the body, he naturally concludes, that bile cannot be prepared unlefs from venal blood; and he alfo infers, that the equal and undifturbed current of blood in the veins is favourable to the fecretion; but the circumftances of the prefent cafe, in which bile was fecreted by an artery, prove, he thinks, the fallacy of this reafoning. He regrets that only fo fmall a quantity of this bile could be collected from the gall bladder; as, furely, it was, he obferves, very defirable to afcertain more accurately how far the qualities of this curioufly-prepared fluid refembled common bile.

That the fluid fecreted by the liver was not, in this cafe, deficient in quantity, appears to him fufficiently evident. If the gall bladder had not fuffered occafional repletion, he thinks it would have been found in a ſtate of greater contraction. Some bile, he obſerves, had eſcaped from the divided gall-ducts, in removing the ſtomach and duodenum, before the uncommon termination of the vena portarum was diſcovered; and a conſiderable quantity of this fluid, he adds, would be required to give ſo deep a tint, as in this caſe was viſible, to the alimentary matter.

He ſuppoſes, therefore, that the empty ſtate of the gall bladder was the effect of accident, and not of deficient ſecretion by the liver. The bulk and well-nouriſhed ſtate of the body do, he thinks, demonſtrate that there was no defect in the functions of the chylopoetic organs.

But it will probably be inquired, from what cauſe the death of the child originated. It may, our author obſerves, be ſuſpected that the mal-formation of the liver contributed to its deceaſe; and particularly as no derangement of any vital organ could be diſcovered. Yet if it be conſidered how frequently children die from nervous irritation, or fever, the probability of this ſuſpicion is, he thinks, diminiſhed. The circumſtances of the caſe, he obſerves, may impreſs others with contrary ſentiments; but he himſelf will remain ſatisfied with with having faithfully defcribed the appearances of the body, and having offered thofe remarks which he thinks deducible from them.

The appearances defcribed in this cafe are reprefented in two plates, for which we muft refer our readers to the work itfelf.

The peculiarity of the next cafe, related by our author, confifts in an uncommon formation of the alimentary canal. The fubject of it was a boy, whofe body was brought to him for diffection; it meafured four feet three inches in length; but was well formed, and had moderately large limbs, but flabby, as if wafted by recent difeafe.

Upon opening the abdomen, which was enormoufly fwoln, there appeared a more than ordinary extent of large inteftines, in a ftate of great diftention.

The diameter of the canal meafured about three inches, and its dimenfions were nearly equal in every part.

The matter, we are told, with which it was turgid was of a greyifh colour, of a pulpy confiftence, having little fœtor, and quite unlike the ufual fœcal contents of the large inteftines.

The length of the colon, Mr. Abernethy

4 obferves, obſerves, was uncommon; for after having, as uſual, aſcended to the right hypochondrium, it was reflected downwards, even into the pelvis; it then, it ſeems, reaſcended to the left hypochondrium, and afterwards purſued its uſual courſe.

After turning aſide this large volume of inteſtine, to examine the other parts of the alimentary tube, our author was ſurpriſed to diſcover that the ſubject contained ſcarcely any ſmall inteſtines. Theſe viſcera, with the ſtomach, lay, he obſerves, in a perfectly collapſed ſtate; and their texture was ſo extremely tender, that they were torn even by a gentle examination. The duodenum, jejunum, and ileum, when detached from the body, and extended, meaſured, it ſeems, only two feet in length, whilſt the extent of the large inteſtines exceeded four feet.

The utmoſt length of the inteſtinal tube, in this ſubject, was little more than ſix feet, whereas it ſhould, it is obſerved, have been about twenty-ſeven feet, had it borne the ordinary proportion to the length of the body.

Mr. Abernethy diſtended and dried this curious alimentary canal, and ſtill has it in preſervation.

As

As the ſmall inteſtines meaſured only two feet in length, this extent, he obſerves, was doubtleſs inſufficient for the preparation and abſorption of chyle; theſe proceſſes muſt therefore, he thinks, have been, in a great degree, performed by the large inteſtines.

The form and ſtature of the boy, our author remarks, ſhow that nutrition was not ſcantily ſupplied; he died, he thinks, evidently from a want of inteſtinal evacuation. Whether the unuſual ſtructure of the canal contributed to the production of diſeaſe, cannot, he obſerves, be readily determined; it appears, however, he adds, very probable that uncommonly formed parts, although capable of ſupporting life, may be leſs adapted to ſuſtain the derangement of functions conſequent to diſeaſe.

